# The tryptophan catabolite or kynurenine pathway in COVID-19 and critical COVID-19: a systematic review and meta-analysis

**DOI:** 10.1186/s12879-022-07582-1

**Published:** 2022-07-15

**Authors:** Abbas F. Almulla, Thitiporn Supasitthumrong, Chavit Tunvirachaisakul, Ali Abbas Abo Algon, Hussein K. Al-Hakeim, Michael Maes

**Affiliations:** 1 Department of Psychiatry, Faculty of Medicine, Chulalongkorn University, Bangkok, Thailand; 2 Medical Laboratory Technology Department, College of Medical Technology, The Islamic University, Najaf, 31001 Iraq; 3 Iraqi Education Ministry, Najaf, Iraq; 4 Department of Chemistry, College of Science, University of Kufa, Kufa, Iraq; 5 Department of Psychiatry, Medical University of Plovdiv, Plovdiv, Bulgaria; 6 Department of Psychiatry, IMPACT Strategic Research Centre, Deakin University, Geelong, VIC Australia

**Keywords:** COVID-19, SARS-Cov-2, Tryptophan catabolites, Inflammation, Oxidative stress

## Abstract

**Background:**

Coronavirus disease 2019 (COVID-19) is accompanied by activated immune-inflammatory pathways and oxidative stress, which both induce indoleamine-2,3-dioxygenase (IDO), a key enzyme of the tryptophan (TRP) catabolite (TRYCAT) pathway. The aim of this study was to systematically review and meta-analyze the status of the TRYCAT pathway, including the levels of TRP and kynurenine (KYN) and the activity of IDO, as measured by the ratio of KYN/TRP.

**Methods:**

This systematic review searched PubMed, Google Scholar, and Web of Sciences and included 14 articles that compared TRP and tryptophan catabolites (TRYCATs) in COVID-19 patients versus non-COVID-19 controls, as well as severe/critical versus mild/moderate COVID-19. The analysis was done on a total of 1269 people, including 794 COVID-19 patients and 475 controls.

**Results:**

The results show a significant (p < 0.0001) increase in the KYN/TRP ratio (standardized mean difference, SMD = 1.099, 95% confidence interval, CI: 0.714; 1.484) and KYN (SMD = 1.123, 95% CI: 0.730; 1.516) and significantly lower TRP (SMD = − 1.002, 95%CI: − 1.738; − 0.266) in COVID-19 versus controls. The KYN/TRP ratio (SMD = 0.945, 95%CI: 0.629; 1.262) and KYN (SMD = 0.806, 95%CI: 0.462; 1.149) were also significantly (p < 0.0001) higher and TRP lower (SMD = − 0.909, 95% CI: − 1.569; − 0.249) in severe/critical versus mild/moderate COVID-19. No significant difference was detected in kynurenic acid (KA) and the KA/KYN ratio between COVID-19 patients and controls.

**Conclusions:**

Our results indicate increased activity of the IDO enzyme in COVID-19 and severe/critical patients. The TRYCAT pathway is implicated in the pathophysiology and progression of COVID-19 and may signal a worsening outcome of the disease.

**Supplementary Information:**

The online version contains supplementary material available at 10.1186/s12879-022-07582-1.

## Background

Infection with severe acute respiratory syndrome coronavirus 2 (SARS-CoV-2) may cause coronavirus disease 2019 (COVID-19) [1]. Some COVID-19 patients may experience acute respiratory distress or even severe acute respiratory syndrome (SARS), which may necessitate admission to an intensive care unit [1, 2]. SARS can also cause organ failure and death, especially in older people and people with type 2 diabetes mellitus (T2DM), high blood pressure, heart disease, stroke, dementia, obesity [1–3], and a high body mass index [4].

COVID-19 is characterized by activated immune-inflammatory pathways and, in some cases, hyperinflammation [5, 6]. Most importantly, during SARS-CoV-2 infection, the cytokine network is activated, with elevated levels of many pro-inflammatory cytokines such as interleukin (IL)-1β, IL-18, IL-6, tumor necrosis factor (TNF)-α, and interferon (IFN)-γ [7–10]. Mild COVID-19 may progress into SARS with pneumonia (and lowered oxygen saturation and lung lesions on chest computerized tomography scan), intravascular coagulation, multisystem failure, and death if these pro-inflammatory cytokines are overproduced during a cytokine storm [2, 7, 8]. Profound tissue damage, even extending to organ failure, may be the consequence of enduring increases in IFN-γ secretion [11]. COVID-19 is accompanied by increased production of reactive oxygen species (ROS) and ensuing oxidative damage, contributing to severe COVID-19 [12–14].

During infection, increased levels of IFN-γ, IL-1β, IL-6, and ROS may induce indoleamine-2,3-dioxygenase (IDO), which activates the catabolism of tryptophan (TRP), thereby lowering serum TRP and increasing tryptophan catabolites (TRYCATs), including kynurenine (KYN), 3-OH-kynurenine (3HK), kynurenic acid (KA), quinolinic acid (QA), and xanthurenic acid (XA) [15]. Activation of the TRYCAT pathway protects against hyperinflammation and microbial invasion by different processes including scavenging ROS, TRP starvation, and negative immunoregulatory effects [15, 16].

Furthermore, some TRYCATs, such as XA and KA, have antioxidant properties [17], whereas KYN, KA, XA, 3HK, and QA have negative immune regulatory effects, such as inhibiting IFN-γ production [16, 18]. Nonetheless, following overproduction of TRYCATs, several detrimental consequences may appear, including oxidative stress, immune activation, and neurotoxic effects [19–25].

**Fig. 1:**
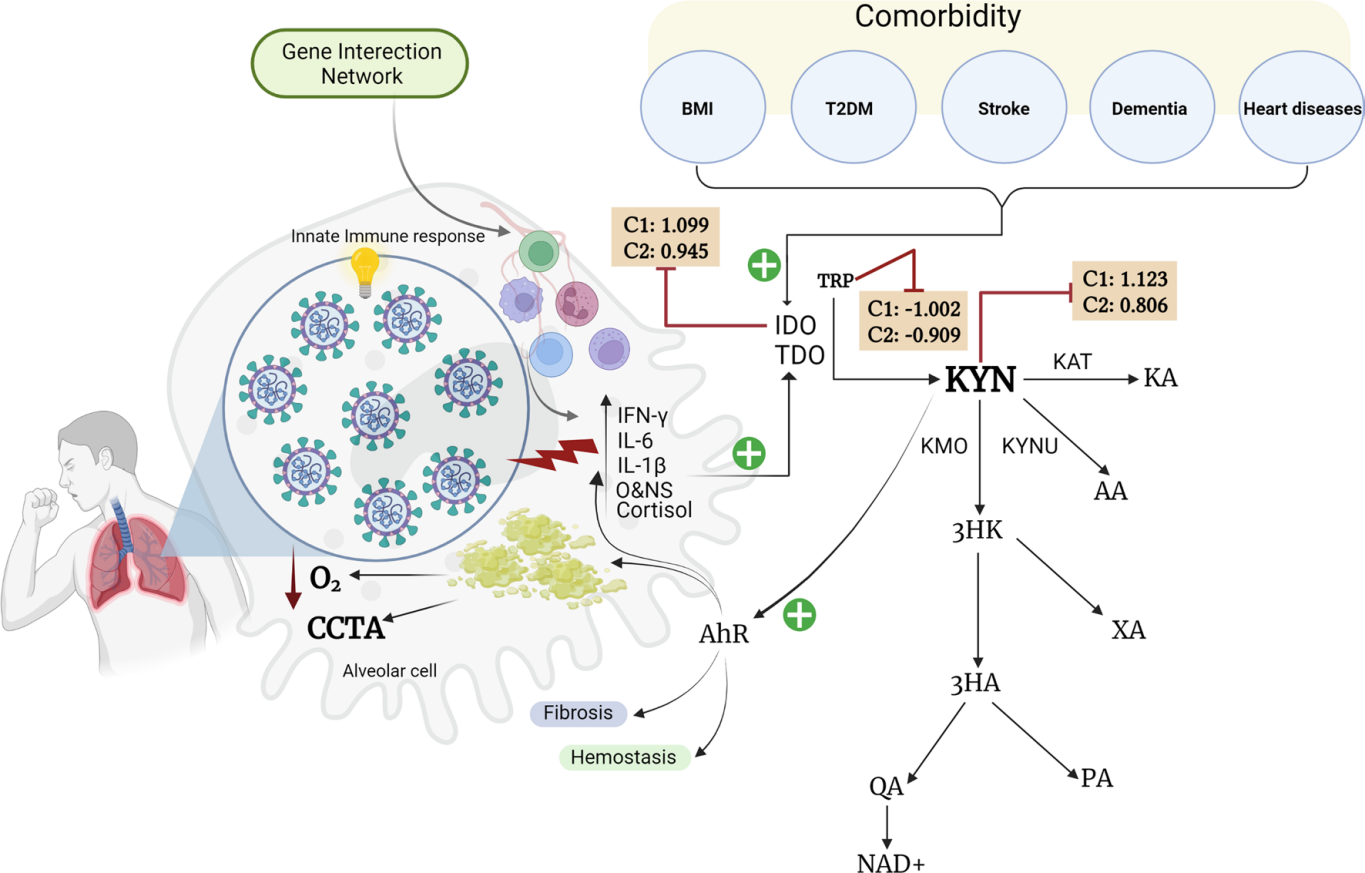
Summary of TRYCAT pathway in COVID-19. *BMI* body mass index, *C* cohort, *T2DM* type 2 diabetes mellitus, *IFN-γ* interferon-Gamma, *IL-6* interleukin 6, *IL-1β* interleukin-1 beta, *O&NS* oxidative and nitrosative stress, *O2* oxygen, *CCTA* chest computed tomography abnormalities, *AhR* aryl hydrocarbon receptor, *IDO* indoleamine 2,3 dioxygenase, *TDO* tryptophan 2,3-dioxygenase, *KAT* kynurenine aminotransferase, *KMO* kynurenine 3-monooxygenase, *KYNU* kynureninase, *TRP* tryptophan, *KYN* kynurenine, *KA* kynurenic acid, *3HK* 3-hydroxykynurenine, *AA* anthranilic acid, *XA* xanthurenic acid, *3HA* 3-hydroxyanthranilic acid, *PA* picolinic acid, *QA* quinolinic acid, *NAD + * nicotinamide adenine dinucleotide. Created with BioRender.com

In COVID-19, some authors reported increased activity of the TRYCAT pathway as indicated by lowered TRP and increased KYN levels and an increased KYN/TRP ratio [26–28], which reflects IDO activity [29]. Figure 1 shows the possible role of the TRYCAT pathway in COVID-19. Probably, the IDO enzyme, which is the first and rate-limiting enzyme of the TRYCAT pathway, is induced in COVID-19 by increased levels of IFN-γ, IL-1, IL-6, TNF-α, and ROS [15]. Moreover, stimulation of the aryl hydrocarbon receptor (AhR) by coronaviruses and IDO-induced KYN levels may cause the “systemic aryl hydrocarbon receptor activation syndrome” (SAAS), which aggravates hyperinflammation, hypercoagulation, and organ injuries [30]. It was hypothesized that TRYCAT pathway activation may worsen COVID-19 and probably decrease the patient's recovery potential [26, 31]. Nevertheless, no systematic review and meta-analysis were conducted on COVID-19 and severe/critical COVID-19 to examine whether the TRYCAT pathway is activated.

Hence, the purpose of the current study was to systematically review and meta analyze the TRP and TRYCAT results in COVID-19 patients versus controls and severe/critical versus mild/moderate COVID-19.

## Methods

The current meta-analysis was in compliance with the standards of Preferred Reporting Items for Systematic Reviews and Meta-Analyses (PRISMA) 2020 [32], the guidelines of the Cochrane Handbook for Systematic Reviews and Interventions [33], and the Meta-Analyses of Observational Studies in Epidemiology (MOOSE). In the present meta-analysis, we examine TRP and TRYCAT levels. We also used the KYN/TRP and (KA + KYN)/TRP ratios to measure the activity of the IDO enzyme and the KA/KYN and KA/(KYN + TRP) ratios to measure the activity of the kynurenine aminotransferase (KAT) enzyme.

### Search strategy

The search for literature started on December 15th, 2021 and ended on December 31st, 2021, when all the required data were collected. We entered specific Mesh terms and keywords in electronic databases to find related articles in PubMed/MEDLINE, Google Scholar, and Web of Science. These terms and keywords, focused on TRP, TRYCATs and COVID-19, are shown in the Additional file 1: Table S1. To ensure that we included all the related articles, we also searched the reference lists of previous reviews and grey literature.

### Eligibility standards

We included published papers in peer-reviewed journals and written in the English language as the main criteria for selecting articles. However, we also reviewed manuscripts published in other languages such as Thai, French, Spanish, German, Italian, and Arabic. Inclusion criteria were: (a) observational case–control and cohort studies that quantified the concentrations of TRP and TRYCATs in serum, plasma, cerebrospinal fluid (CSF), and brain tissues of patients who showed a positive real-time polymerase chain reaction (RT-PCR) test for SARS-CoV-2 and were either symptomatic or asymptomatic; and (b) studies reporting data in a control group consisting of healthy people, previously infected or recovered patients, or a subgroup of mild/moderate COVID-19 patients; and (c) the results are reported as quantitative scores with mean and standard deviation (SD) or standard error of the mean (SEM). We excluded the following studies: (a) systematic and narrative reviews and meta-analysis studies; (b) duplicate studies as well as animal and genetic studies; (c) articles that used other media, including saliva; and (d) the authors did not show mean and SD/SEM of the measured biomarkers or any other mean to estimate these values. When the authors presented geometric means, medians (interquartile range, range), or represented data as a graph, we sent emails to request the mean ± SD in the study groups. Without response from the authors, we used the estimation method described by Wan and Wang [34] to compute mean ± SD from the median with either interquartile range or range. In addition, Web Plot Digitizer (https://automeris.io/WebPlotDigitizer/) was also used to get quantitative information from a graph.

### Primary and secondary outcomes

The primary outcome is IDO activity, which we assessed through the KYN/TRP and (KYN + KA)/TRP ratios [35] in COVID-19 patients versus controls. Secondary outcomes were the KA/KYN and KA/(KYN + TRP) indices which reflect KAT enzyme activity. The TRP and TRYCATs data were not only compared between people with COVID-19 and people who did not have COVID-19 (study cohort 1), but they were also compared between people with severe/critical COVID-19 (some of whom died from it) and people with mild/moderate COVID-19 (study cohort 2).

### Screening and data extraction

The first author (AA) performed an initiatory review by evaluating the titles and abstracts to ensure which papers were eligible to be included. Consequently, eligible full-text articles were downloaded after removing some publications according to the predetermined exclusion criteria. All required data extracted from the articles were entered in a predefined excel spreadsheet file made for this project, including researcher's names, publication date, quantitative data of TRP and TRYCATs, the number of the participants either as a COVID-19 or control groups, demographic data such as age (expressed as mean ± SD), male/female count, type of sample, serum or plasma, severity level, country latitude in which the study was conducted, and quality scores of the studies (see below).

Furthermore, all extracted data in the excel spreadsheet were scrutinized by the second author (TS) immediately after the first author finalized entering the data. The last author (MM) was consulted in the case of controversial results. The last author slightly adjusted the “immune confounder scale (ICS)” published previously [36–38] to estimate the methodological quality of TRYCAT studies. This ICS and the related repoint checklist are shown in Additional file 1: Table S2. These scores estimate key quality data such as sample size, covariate control and the time of sampling. The best methodological quality is obtained when the ICS score is close to 10 with the overall score ranging from 0 to 10. The redpoints score scale mainly focuses on the poor adjustment of the key confounders, which may cause biased TRYCATs results (either due to biological or analytical variation), along with an uncontrolled study design. The range of the score scale is from 0 to 26 with values close to 26 indicating poor control and quality.

### Data analysis

We employed the CMA V3 software to conduct the current meta-analysis and we followed the PRISMA guidelines [39]. The presence of TRYCATs in at least three studies was the determinant for conducting a meta-analysis. The biomarker’s outcomes as assessed in our systematic review and meta-analysis are displayed in Table 1. By calculating the mean values of the markers in their respective profiles (e.g. KYN/TRP ratio) and assuming dependency, we compared the synthetic scores indicating these profiles in COVID-19 patients (or subgroups) versus their controls. In the meta-analysis, IDO activity was estimated by specifying the direction of the effect size of KYN as positive (favoring COVID-19) and TRP as negative. Furthermore, KAT activity was estimated by entering KA with a positive direction (thus favoring COVID-19) and TRP and/or KYN with a negative direction in the meta-analysis. A restricted maximum-likelihood random-effects model was utilized based on our hypothesis that the included studies have different characteristics. The standardized mean difference (SMD) with 95% confidence intervals (95% CI) was computed as the indicator for the effect size. We considered the results to be statistically significant when p < 0.05 (two-tailed tests). SMD values of 0.8, 0.5, and 0.2 indicate large, moderate, and small effect sizes, respectively [40]. Heterogeneity was examined by tau-squared values as mentioned previously and we also computed the Q and I^2^ metrics [41, 42]. We also used the leave-one-out approach to conduct sensitivity analyses to assess the robustness of the pooled combined meta-analysis effects and between-study heterogeneity. We assessed possible differences in TRP and TRYCATs between serum and plasma [37] by considering these subgroups as a unit of analysis. We compared the effects at different study levels and ran the meta-analysis across subgroups. We assessed the impact of small study effects, including publication bias, using the conventional fail-safe N approach, Kendall tau with continuity correction (using one-tailed p-values), and Egger's regression intercept (using one tailed p-values). When Egger's linear regression test indicates substantial asymmetry, we estimate the modified effect size after accounting for the impacts of missing studies using Duval and Tweedie's trim-and-fill approach. We conducted random-effect meta-regression analyses to estimate the impact of covariates including age, sex, country latitude in which the study was conducted, type of medium, severity of illness and quality scores of the studies.

**Table 1:** The number of COVID-19 patients and studies included in the meta-analyses and the side of standardized mean difference (SMD) and the 95% confidence intervals with respect to the zero SMD

Outcome profiles	n studies	Side of 95% confidence intervals				Patient Cases	Control Cases	Total number of participants
		< 0	Overlap 0 and SMD < 0	Overlap 0 and SMD > 0	> 0			
Cohort 1: COVID-19 patients versus non-COVID-19 control								
KYN/TRP	10	1	0	2	7	329	475	804
KYN	8	0	1	0	7	285	419	704
TRP	7	5	1	1	0	275	409	684
KA	4	0	1	2	1	113	94	207
KA/(KYN + TRP)	8	1	1	4	2	285	419	704
(KYN + KA)/TRP	10	1	0	3	6	329	475	804
KA/KYN	8	3	3	2	0	285	419	704
Cohort 2: severe/critical COVID-19 patients versus mild/moderate COVID-19 patients								
KYN/TRP	9	0	0	1	8	270	503	773
KYN	6	0	0	3	3	184	399	583
TRP	5	3	2	0	0	153	282	435

*KYN* kynurenine, *TRP* tryptophan, *KA* kynurenic acid

## Results

### Search findings

**Fig. 2:**
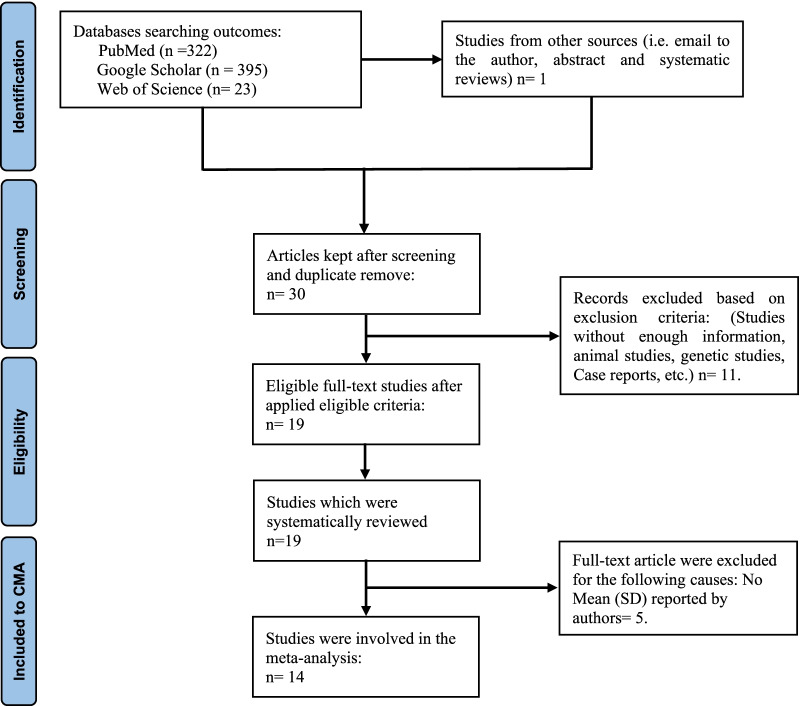
Prisma flow chart

During the selection process, 30 articles were investigated in the current study based on the keywords shown in Additional file 1: Table S1. The detailed information related to the inclusion–exclusion criteria of the research papers and the outcomes of our search process is presented in the PRISMA flowchart shown in Fig. 2. Nineteen full-text papers were eligible for the systematic review after 11 records were removed from the initial number of articles. Finally, the meta-analysis involved 14 articles as 5 papers were excluded for reasons listed in Additional file 1: Table S3 [26, 27, 31, 43–53].

In this meta-analysis, we considered case–control and retrospective studies. This study examined 804 subjects in study cohort 1 (329 COVID-19 patients and 475 non-COVID-19 controls), and 773 individuals in the second cohort (involving 270 severe/critical COVID-19 patients and 503 mild/moderate COVID-19 patients). As shown in Additional file 1: Table S3, we excluded 5 studies from the meta-analysis. In Cohort 1, TRYCATs were examined in plasma in 6 studies while 4 studies used serum, whereas in the second cohort, 6 studies were based on plasma and 3 used serum. Liquid chromatography-mass spectrometry (LC–MS) was used in 5 studies, while 3 studies utilized liquid chromatography and two mass spectrometry (LC–MS/MS). In two studies, ultra-high-performance liquid-chromatography-mass spectrometry (UHPLC-MS) and high-performance liquid chromatography were employed and the remaining studies used liquid chromatography–high-resolution mass spectrometry (LC–HRMS) and liquid chromatography-UV detection (LC-UV). All the included studies were conducted on patients who showed positive COVID-19 with varying degrees of severity. In the control group, some authors reported that they never got an infection with COVID-19 and others just mentioned they were not infected.

Overall, within 14 eligible studies, there were 794 COVID-19 patients (329 in case control studies and 465 in retrospective studies) and 475 non-COVID-19 controls. The ages of the participants were between 40 and 95 years old. Brazil, USA, Latvia, Canada, France, China, Mexico, Sweden, Spain, and Italy each contributed one study, while Australia and Austria each contributed two studies. However, most participants were from Italy due to the large sample size. Additional file 1: Table S4 shows the median (min–max) ICS score, namely redpoint and quality scores which equaled 12.5 (min = 6, max = 17) and 3 (min = 3, max = 7), respectively.

### COVID-19 versus controls

#### The primary outcome variables KYN/TRP and (KYN + KA)/TRP ratio

**Fig. 3:**
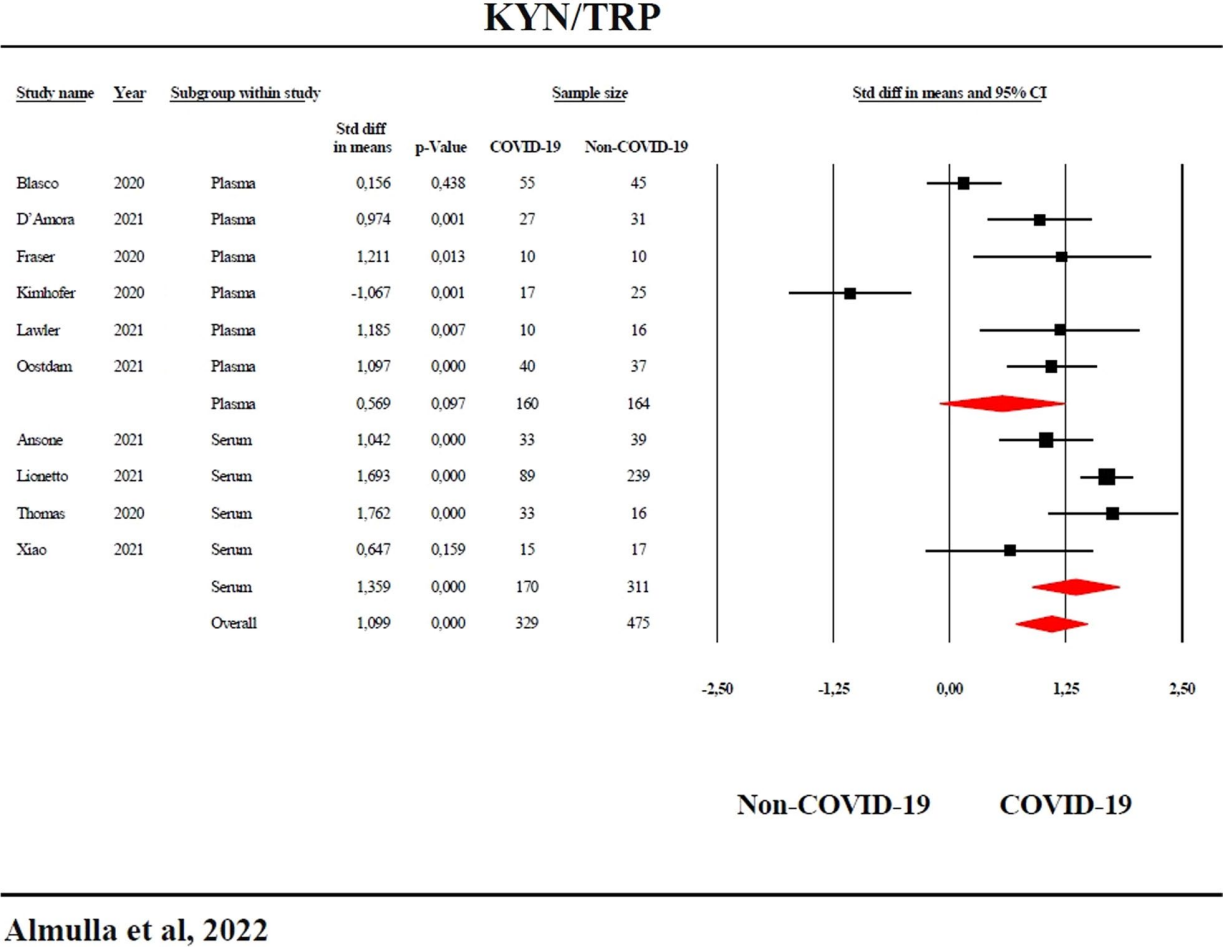
Forest plot with the results of a meta-analysis performed on the kynurenine/tryptophan (KYN/TRP) ratio in COVID-19 patients versus non-COVID-19 controls

The results of the systematic review on KYN/TRP in COVID-19 are shown in Table 1. We found that out of the ten included studies, the 95% CI for 7 (4 serum, 3 plasma) were entirely on the positive side of zero, while only one (plasma) study was totally on the negative side of zero. The two other studies showed 95% CI that overlapped with zero but with SMD values that were greater than zero. Figure 3 shows the forest plot of KYN/TRP in COVID-19 patients versus non-COVID-19 controls. We performed subgroup analyses to examine the high heterogeneity as indicated by elevated values of τ^2^. These results showed a trend toward a possible difference (p = 0.059) between serum and plasma. The serum results displayed a huge and significant effect size between COVID-19 and controls, whereas the plasma findings were non-significant (Table 2). In addition, in serum, the heterogeneity was lower as compared with plasma. We found 3 missing studies on the left side and imputation of these missing studies lowered the SMD to 0.573 (95% CI: 0.003; 1.144), although still significant. Serum results did not show any bias; while there was 1 missing study in plasma, and after imputation, the SMD decreased to 0.462 (95% CI: − 0.155; 1.080).

Table 1 shows that in 6 studies (out of 10) the 95% CI were totally on the right side of zero and that only one study showed that the CI were completely on the left side of zero. The 95% CI of the other 3 studies crossed the zero line, but all showed SMD values that were greater than zero. Table 2 shows there is a significant difference in (KYN + KA)/TRP ratio between COVID-19 patients and controls with a large effect size of SMD = 0.789. Additional file 2: Fig. S1 displays the forest plot of the (KYN + KA)/TRP ratio. Table 3 shows no evidence of publication bias, although there were two missing studies on the left side and a somewhat lower adjusted SMD after imputation (SMD = 0.579).

**Table 2:** Results of meta-analysis performed on several outcome variables of the tryptophan catabolite (TRYCAT) pathway

Outcome feature sets	n	Groups	SMD	95% CI	z	p	Q	df	p	I^2^ (%)	τ^2^	Τ
Cohort 1: COVID-19 patients versus non-COVID-19 control												
KYN/TRP	10	Overall	1.099	0.714; 1.484	5.594	< 0.0001	85.310	9	< 0.0001	89.450	0.616	0.785
	4	Serum	1.359	0.889;1.829	5.666	< 0.0001	9.104	3	0.028	67.049	0.145	0.390
	6	Plasma	0.569	− 0.103; 1.241	1.661	0.097	38.093	5	< 0.0001	86.874	0.591	0.769
	Q = 3.568, df = 1, p = 0.059											
KYN	7	Overall	1.123	0.730; 1.516	5.595	< 0.0001	29.745	7	< 0.0001	76.467	0.221	0.470
TRP	7	Overall	− 1.002	− 1.738; − 0.266	− 2.668	0.008	88.926	6	< 0.0001	93.253	0.892	0.944
KA	4	Overall	0.164	− 0.120; 0.449	1.133	0.257	6.947	3	0.074	56.816	0.128	0.358
	2	Serum	0.649	0.170; 1.129	2.653	0.008	0.617	1	0.432	0.000	0.000	0.000
	2	Plasma	-0.098	− 0.451;0.255	− 0.546	0.585	0.275	1	0.600	0.000	0.000	0.000
	Q = 6.055, df = 1, p = 0.014											
KA/(KYN + TRP)	10	Overall	0.297	0.089;0.506	2.794	0.005	40.957	9	< 0.0001	78.026	0.255	0.505
(KYN + KA)/TRP	10	Overall	0.789	0.261;1.318	2.926	0.003	87.188	9	< 0.0001	89.678	0.624	0.790
KA/KYN	8	Overall	− 0.398	− 0.967; 0.170	-1.373	0.170	66.867	7	< 0.0001	89.531	0.569	0.754
Cohort 2: severe/critical COVID-19 patients versus mild/moderate COVID-19 patients												
KYN/TRP	8	Overall	0.945	0.629; 1.262	5.848	< 0.0001	25.776	8	0.001	68.964	0.151	0.389
KYN	6	Overall	0.806	0.462; 1.149	4.593	< 0.0001	13.343	5	0.020	62.528	0.108	0.328
TRP	5	Overall	− 0.909	− 1.569; − 0.249	− 2.699	0.007	28.165	4	< 0.0001	85.798	0.462	0.680

*KYN* kynurenine, *TRP* tryptophan, *KA* kynurenic acid

**Table 3:** Results on publication bias

Outcome feature sets	Fail safe n	Z Kendall’s τ	p	Egger’s t test (df)	p	Missing studies (side)	After adjusting
Cohort 1: COVID-19 patients versus non-COVID-19 control							
KYN/TRP (overall)	10.499	0.089	0.464	0.989 (8)	0.175	3 (left)	SMD = 0.573 0.003; 1.144)
KYN/TRP (serum)	11.244	0.679	0.248	1.223 (2)	0.172	0	
KYN/TRP (plasma)	4.373	0.563	0.286	0.347 (4)	0.372	1 (left)	SMD = 0.462 (− 0.155; 1.080)
KYN (overall)	11.647	0.494	0.310	0.269 (6)	0.398	2 (left)	SMD = 0.961 (0.584; 1.338)
TRP (overall)	− 11.267	0.150	0.440	0.947 (5)	0.193	1 (Right)	SMD = − 0.817 (− 1.618; − 0.017)
KA (overall)	2.478	0.000	0.500	0.720 (2)	0.273	0	
KA/(KYN + TRP) (Overall)	1.673	1.162	0.122	0.549 (8)	0.298	1 (Left)	SMD = 0.023 (− 0.358;0.405)
(KYN + KA)/TRP (overall)	9.818	0.804	0.210	1.213 (8)	0.129	2 (Left)	SMD = 0.579 0.0007; 1.157)
KA/KYN (Overall)	− 5.376	0.247	0.402	1.598 (6)	0.080	0	
Cohort 2: severe/critical COVID-19 patients versus mild/moderate COVID-19 patients							
KYN/TRP (Overall)	10.226	1.251	0.105	2.362 (7)	0.025	1 (Left)	SMD = 0.876 (0.562; 1.189)
KYN (overall)	7.758	0.187	0.425	0.129 (4)	0.451	0	
TRP (overall)	-5.887	1.959	0.025	5.897 (3)	0.004	0	

Table 1 and Additional file 2: Fig. S2 display the forest plot of TRP in COVID-19. Table 2 shows an overall significant decrease in TRP in COVID-19 with a high effect size (SMD = − 1.002). Although there were no significant differences (p = 0.404) between plasma and serum, serum TRP was significantly decreased in COVID-19 (SMD = − 1.216), whereas plasma TRP did not show significant differences. Table 3 shows no evidence of publication bias, although Duval and Tweedie’s trim and fill showed one missing value on the right side and imputation yielded an adjusted SMD of − 0.817.

Table 1 shows that out of 8 KYN studies, the 95% CI of 7 studies was completely on the right side of zero, while one CI intersected with zero. Additional file 2: Fig. S3 and Table 2 show the KYN results, indicating a highly significant increase in KYN in COVID-19 (SMD = 1.123). Duval and Tweedie’s trim and fill showed two missing studies on the left side  and imputing these studies yielded a slightly decreased effect size (SMD = 0.961, 95% CI: 0.584; 1.338), which was still significant.

### Secondary outcome variables

#### KA and KA ratios 

Table 1 and 2 and Additional file 2: Fig. S5 show the KA, KA/KYN and KA/(KYN + TRP) results. There was a significant increase with a small effect size (SMD = 0.297) in KA/(KYN + TRP) in COVID-19 patients as compared with controls. However, after imputing one missing study, the SMD decreased to 0.023 and was no longer significant (Table 3).

KA results were obtained in 4 studies. Table 1 and Additional file 2: Fig. S6 show that 3 studies intersected with zero, with 2 studies showing SMD values greater than zero and one study less than zero, while one study showed 95% CI, which were completely on the right side of zero. There was a high heterogeneity when serum and plasma were combined, with a significant difference (p = 0.014) between both media. Therefore, we conducted a subgroup analysis showing that the results in serum contradicted those in plasma (see Table 2).

### Severe/critical COVID-19 versus mild/moderate COVID-19

**Fig. 4:**
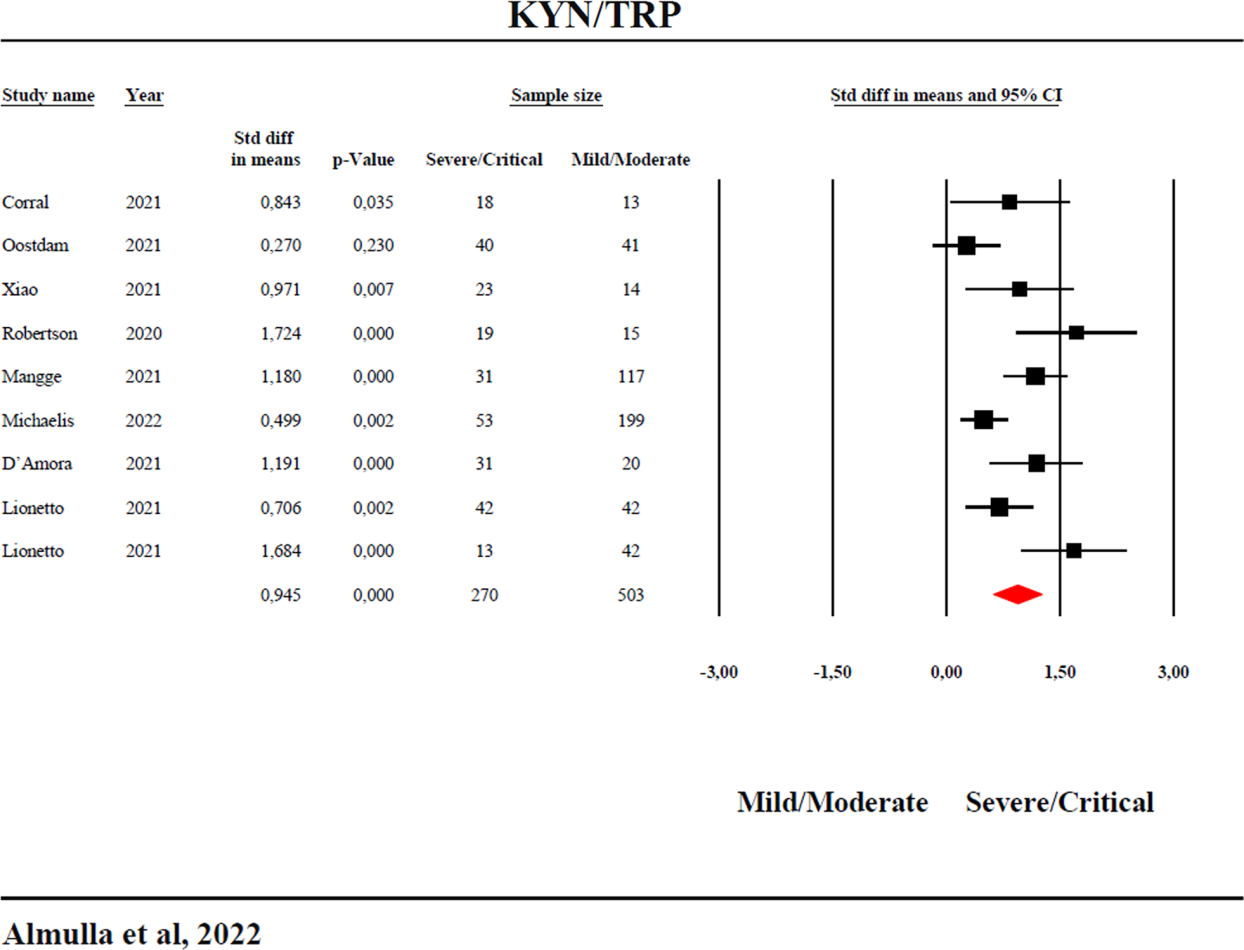
Forest plot with the results of a meta-analysis performed on the kynurenine/tryptophan (KYN/TRP) ratio in severe/critical COVID-19 versus mild/moderate COVID-19

Table 1 shows that all 95% CI of the cohort 2 studies reporting on severe/critical versus mild/moderate COVID-19 were completely on the right side of zero (favoring severe/critical patients), except 1 study which crossed zero. There is a significant difference in the KYN/TRP ratio between severe/critical versus mild/moderate COVID-19 with a huge effect size favoring severe/critical COVID-19 (SMD = 0.945). Figure 4 shows the forest plot of the KYN/TRP ratio between severe/critical versus mild/moderate patients. Publication bias with one missing study was detected and the adjusted SMD was slightly less than the observed but was still significant with a high impact size (SMD = 0.876). Additional file 2: Fig. S7 and Table 2 show significantly lowered TRP in severe/critical COVID-19 as compared with mild/moderate COVID-19 with a large effect size (SMD = − 0.909). Table 2 and Additional file 2: Fig. S8 show an overall significant difference in KYN levels between severe/critical and mild/moderate COVID-19 with a large effect size (SMD = 0.806). Table 3 did not show evidence of publication bias in the KYN data in critical COVID-19.

### Meta-regression analysis

The meta-regression results are shown in Additional file 1: Table S5 indicating that the type of medium affected the KYN/TRP (p = 0.047) and (KYN + KA)/TRP (p = 0.051) ratios, and also KA (p = 0.006). Furthermore, sample size had a significant (p = 0.022) effect on the KA/KYN ratio, and disease severity had a significant (p = 0.003) effect on the KA/(KYN + TRP) ratio.

## Discussion

### IDO and KAT in COVID-19

The first major findings of this systematic review and meta-analysis are that (a) the KYN/TRP ratio is significantly increased in COVID-19 patients compared to non-COVID-19 controls with a high effect size; and (b) the KYN/TRP ratio is dramatically increased in severe/critical COVID-19 as compared with mild/moderate COVID-19 again with a large effect size. Importantly, the severe/critical COVID-19 patient samples included in this study mainly consist of critical patients who did not survive, and therefore, our results also suggest that an increased KYN/TRP ratio is associated with death due to COVID-19. These results indicate that IDO activity and the TRYCAT pathway are upregulated in COVID-19 and that it predicts critical disease and non-survival. The most probable cause of IDO enzyme activation in COVID-19 is the increased level of pro-inflammatory cytokines including IFN-γ, IL-1β and IL-6 [54, 55] and activated oxidative stress pathways [56], which both potently stimulate IDO [26, 57].

Further analyses showed that the changes in the KYN/TRP ratio are attributable to significant increases in KYN and decreases in TRP in COVID-19, again with large effect sizes. These results extend the findings of previous studies which showed associations between the severity of COVID-19 and increases in the KYN/TRP ratio and KYN and decreases in TRP [26, 52, 53]. Thus, TRYCAT pathway activation appears to contribute to a fatal course of the disease [58–60].

The second major finding of this study is that the KA/KYN ratio did not show a significant difference between COVID-19 patients as compared to non-COVID-19 controls, indicating no significant changes in KAT activity. Serum KA was significantly increased in COVID-19 with a medium effect size (0.649), whereas in plasma a non-significant inverse association was found. There is insufficient data to perform meta-analysis on other ratios reflecting kynurenine 3-monooxygenase (KMO) and kynureninase (KYNU) activity. In this respect, Lawler et al., reported elevated levels of 3HK and QA in patients with COVID-19 compared to healthy controls [49]. Likewise, Marin-Corral et al. reported a high level of 3HK in severe/critical COVID-19 patients compared to those with mild/moderate infection [52].

### Role of the TRYCAT pathway in COVID-19

During infection, IDO activation and consequent increased TRYCATs but lowered TRP levels are key components of the innate immune response. First, the TRYCAT pathway has major intrinsic scavenging activities by neutralizing ROS [15]. Moreover, some TRYCATs have antioxidant properties on their own, as for example, 3-hydroxyanthranilic acid (3HA) and 3HK, which are more effective as radical scavengers than tocopherol, and XA, which has antioxidant activity comparable to that of butylated hydroxytoluene (BHT) [15, 17]. By protecting tissues from oxidative damage, KA has adequate antioxidant effects [61, 62]. Second, reduced TRP exerts anti-inflammatory (reduced T cell proliferation and activation, sensitization of apoptosis of activated T cells, and induction of the regulatory phenotype) and antimicrobial (inhibiting the growth of viruses, bacteria and parasites) effects through TRP starvation [63–67]. Third, TRYCATs such as KA, KYN, QA, and XA may have a negative immune-regulatory effect by decreasing IFN-γ production and/or increasing IL-10 production [15, 18]. In addition, KA has potent anti-inflammatory effects, while diminished KA levels may aggravate tissue damage and cell proliferation [68]. IFN-γ-induced stimulation of antigen-presenting cells upregulates the TRYCAT pathway and results in a counter-regulatory effect that preserves homeostasis [69]. Due to the fact that TRYCATs trigger apoptosis in Th-1, but not Th-2, cells, TRYCAT pathway activation may suppress Th-1 cells and promote Th-2 cell survival [70, 71]. As such, TRYCAT pathway activation results in a negative feedback loop to limit ROS production, hyperinflammation, and the Th-1 response [18, 70]. Fourth, some TRYCATs have neuroprotective effects including KA, anthranilic acid (AA) and XA. Thus, KA may inhibit *N*-methyl-d-aspartate (NMDA), kainate glutamate ionotropic, and amino-3-hydroxy-5-methyl-4-isoxazolepropionic acid (AMPA) receptors, and reduce glutamate liberation through attenuating alpha 7 nicotinic acetylcholine receptors [16, 72]. XA inhibits vesicular glutamate transport (VGLUT), synaptic transmission via the NMDAR receptor, and excitatory postsynaptic potentials [73]. Furthermore, AA has neuroprotective effects by blocking the synthesis of neurotoxic TRYCATs such as picolinic acid (PA) and QA from 3HA [74].

Nevertheless, overproduction of some TRYCATs may cause detrimental effects on COVID-19. KA is implicated in deteriorating male COVID-19 patients through affecting the AhR, one of the master regulators of the immune-inflammatory response [75]. In addition, activation of AhR by TRYCATs, mainly KYN, affects immune resistance against viral infections and the airway basal cells of the lung epithelium, which are responsible for tissue repair [50, 76]. Most importantly, coronaviruses activate the same receptor through an IDO-independent mechanism while the IDO-AhR pathway in employed by viruses, bacteria, and parasites to establish infection [30]. Consequently, a positive feedback loop is established between increased TRYCATs levels due to IDO activation and stimulation of the AhR by TRYCATs and coronavirus [30]. Moreover, the AhR may enhance IDO transcription and regulate IDO activity [77]. These processes may result in the SAAS which may result in activated immune-inflammatory pathways (increased M1 cytokines), fibrosis (increased IL-22), thromboembolism (increased tissue factor and plasminogen activator inhibitor-1, consequent multiple organ injuries including brain injuries, and eventually death [30].

### Role of TRYCAT pathway in comorbidities

Some TRYCATs have depressogenic, anxiogenic and neurotoxic effects, and TRYCATs like KYN are increased in neuropsychiatric illness including major depression, anxiety, and psychosis [15, 78]. Some TRYCATs exhibit pro-oxidant properties as evidenced by increased ROS, hydrogen peroxide, and superoxide production, and increased oxidative damage, including lipid peroxidation caused by 3HA, 3HK, and QA [19–25]. TRYCATs such as QA and XA and PA may have direct neurotoxic effects by activating hippocampal NMDAR and causing excitotoxicity with apoptosis and hippocampal shrinkage, thereby inducing neurocognitive impairments [79, 80]. Elevated XA levels may cause severe neuronal damage, apoptosis, mitochondrial dysfunctions, disrupt glutamate transmission, and impair presynaptic transmission caused by NMDAR stimulation [73]. Such effects may contribute to the development of neuropsychiatric disorders such as depression, anxiety and chronic fatigue due to COVID-19 [81]. Indeed, TRYCATs are confirmed to be associated with various mental disorders, including depression, and anxiety [15, 18], somatization and chronic fatigue syndrome [82], cognitive impairments [83], and psychosis [37]. Moreover, some TRYCATs, namely KYN, KA and 3HK are associated with musculoskeletal injuries due to their agonistic effects on the AhR [84–87]. Thus, increased TRYCAT levels could exacerbate the neuro-immune and neuro-oxidative toxicity caused by increased oxidative stress and M1 and Th-1 activation, resulting in comorbid affective disorders [81]. Therefore, it is safe to say that the accumulation of TRYCATs in SARS-CoV2-infected patients may play a role in the neuropsychiatric and cognitive syndromes of long or post-COVID syndrome [88].

Finally, it may be hypothesized that COVID-19-associated TRYCAT pathway activation may aggravate the existing disorders in this pathway in comorbid disorders (obesity, dementia, T2DM, hypertension and heart disease, stroke, chronic obstructive pulmonary disease (COPD) and chronic kidney disease) [1–3]. Indeed, in all those comorbid diseases, the IDO enzyme is activated as indicated by an increased KYN/TRP ratio [89–95]. By inference, when COVID-19 develops in people with those comorbid illnesses, an amplified TRYCAT response may occur, contributing to aggravated toxicity in addition to the consequences of inflammation and oxidative stress.

### TRP and TRYCAT assays in serum and plasma

Another finding of our meta-analysis revealed differences in the TRYCAT levels between COVID-19 patients and controls depending on whether plasma and serum was examined. For example, the results of KYN/TRP ratio in serum were highly significant with a large effect size (1.359), whereas in plasma no significant differences were found. Group analysis performed on the KA studies showed a significant difference in effect size between serum and plasma with serum KA yielding a positive medium effect size (0.649), whereas in plasma a negative effect size was established. Similar results were detected in the associations between TRYCATs (e.g. KYN and KA) and schizophrenia with positive results in serum and often inverse results in plasma [37].

### Limitations

Some limitations of the current systematic review and meta-analysis should be discussed. Not all studies clearly describe the types of medications, the treatment protocol, the relevant comorbidities, and even the vaccination status of the patients. Moreover, non survivors following COVID-19 were sometimes lumped together with survivors. Due to the small sample sizes and paucity of data on some TRYCATs, we were unable to estimate KMO and KYNU activity. Therefore, well-powered studies should be conducted in the different stages of COVID-19 (mild, moderate, severe, critical, and non-survival) to assay serum TRP and a more complete panel of serum TRYCATs.

## Conclusions

Figure 1 summarizes the main findings of this study. The TRYCAT pathway is highly activated in COVID-19 and critical COVID-19 as indicated by increased IDO enzyme activity, which was assessed using the KYN/TRP ratio, and increased KYN but reduced TRP levels. KAT enzyme activity was not altered during COVID-19. TRYCATs probably contribute to the pathophysiology, severity and progression of COVID-19. The PRISMA checklist for all parts of our systematic review report is shown in Additional file 1: Table S6.

## Supplementary Information

**Additional file 1**: **Table S1. **Search sentences and terms used in each database. **Table S2. **Immune cofounder’s scale (ICS) (adapted from Andrés-Rodríguez, et al. 2019). **Table S3.** Studies excluded from the meta-analysis but included in the systematic review. **Table S4. **Characterstics of the studies included in the systematic reviews and meta-analysis. **Table S5**. Results of Meta-regression.

**Additional file 2**. **Figure S1:** Forest plot with the results of a meta-analysis performed on the kynurenine + kynurenic acid / tryptophan (KYN+KA)/TRP ratio in COVID-19 patients versus non-COVID-19 controls. **Figure S2:** Forest plot with the results of the meta-analysis performed on tryptophan (TRP) in COVID-19 patients versus non-COVID-19 controls. **Figure S3:** Forest plot with the results of a meta-analysis performed on kynurenine (KYN) in COVID-19 patients versus non-COVID-19 controls. **Figure S4:** Forest plot with the results of a meta-analysis performed on the kynurenic acid / kynurenine (KA/KYN) ratio in COVID-19 patients versus non-COVID-19 controls. **Figure S5:** Forest plot with the results of the meta-analysis performed on the kynurenic acid / kynurenine + tryptophan (KA/KYN+TRP) ratio in COVID-19 patients versus non-COVID-19 controls. **Figure S6:** Forest plot with the results of a meta-analysis performed on kynurenic acid (KA) in COVID-19 patients versus non-COVID-19 controls. **Figure S7:** Forest plot with the results of the meta-analysis performed on tryptophan (TRP) in severe/critical COVID-19 versus mild/moderate COVID-19. **Figure S8:** Forest plot with the results of a meta-analysis performed on kynurenine (KYN) in severe/critical COVID-19 versus mild/moderate COVID-19.

## Data Availability

The datasets used (excel file) and/or analyzed during the current study are available from MM on reasonable request and once all data are exploited by the authors.

## Abbreviations

TRP: Tryptophan
KYN: Kynurenine
KA: Kynurenic acid
3HK: 3-Hydroxykynurenine
AA: Anthranilic acid
3HA: 3-Hydroxyanthranilic acid
XA: Xanthurenic acid
QA: Quinolinic acid
PA: Picolinic acid
IDO: Indoleamine 2,3 dioxygenase
KAT: Kynurenine aminotransferase
KMO: Kynurenine 3-monooxygenase
KYNU: Kynureninase
COVID-19: Coronavirus disease-2019
SARS-Cov-2: Severe acute respiratory syndrome coronavirus 2
TRYCATs: Tryptophan catabolites
TRYCAT pathway: Tryptophan catabolite pathway
KP: Kynurenine pathway
SMD: Standardized mean difference
IL-6: Interleukin-6
IFN-γ: Interferon-gamma
SAAS: Systemic aryl hydrocarbon receptor activation syndrome
T2DM: Type 2 diabetes mellitus
BMI: Body mass index
AhR: Aryl hydrocarbon receptors
PRISMA: Preferred Reporting Items for Systematic Reviews and Meta-Analyses
MOOSE: Meta-Analyses of Observational Studies in Epidemiology
CSF: Cerebrospinal fluid
RT-PCR: Real time-polymerase chain reaction
SD: Standard definition
IOR: Interquartile range
ICS: Immune confounder scale
CI: Confidence interval
LC–MS: Liquid chromatography–mass spectrometry
LC–MS/MS: Liquid chromatography with two mass spectrometry
UHPLC-MS: Ultra-high-performance liquid-chromatography-mass spectrometry
LC–HRMS: Liquid chromatography–high-resolution mass spectrometry
LC-UV: Liquid chromatography-UV detection
CKD: Chronic kidney disease
α7nAChr: Alpha 7 nicotinic acetylcholine receptor
NMDA: *N*-Methyl-d-aspartate
AMPA: α-Amino-3-hydroxy-5-methyl-4isoxazolepropionic acid
