# Supplementary material for: The tryptophan catabolite or kynurenine pathway in COVID-19 and critical COVID-19: a systematic review and meta-analysis
Source: BMC Infect Dis. 2022 Jul 15;22:615. doi: 10.1186/s12879-022-07582-1 (PMC9284970; doi:10.1186/s12879-022-07582-1)
Supplement: Supplementary file 2 — Additional file 2. Figure S1: Forest plot with the results of a meta-analysis performed on the kynurenine + kynurenic acid / tryptophan (KYN+KA)/TRP ratio in COVID-19 patients versus non-COVID-19 controls. Figure S2: Forest plot with the results of the meta-analysis performed on tryptophan (TRP) in COVID-19 patients versus non-COVID-19 controls. Figure S3: Forest plot with the results of a meta-analysis performed on kynurenine (KYN) in COVID-19 patients versus non-COVID-19 controls. Figure S4: Forest plot with the results of a meta-analysis performed on the kynurenic acid / kynurenine (KA/KYN) ratio in COVID-19 patients versus non-COVID-19 controls. Figure S5: Forest plot with the results of the meta-analysis performed on the kynurenic acid / kynurenine + tryptophan (KA/KYN+TRP) ratio in COVID-19 patients versus non-COVID-19 controls. Figure S6: Forest plot with the results of a meta-analysis performed on kynurenic acid (KA) in COVID-19 patients versus non-COVID-19 controls. Figure S7: Forest plot with the results of the meta-analysis performed on tryptophan (TRP) in severe/critical COVID-19 versus mild/moderate COVID-19. Figure S8: Forest plot with the results of a meta-analysis performed on kynurenine (KYN) in severe/critical COVID-19 versus mild/moderate COVID-19. [file 12879_2022_7582_MOESM2_ESM.docx]

**Additional File 2**

**The tryptophan catabolite or kynurenine pathway in COVID-19 and critical COVID-19: a systematic review and meta-analysis.**

SHORTTITLE: Kynurenine pathway in COVID-19

Abbas F. Almulla, Ph.D.^a,b^ , Thitiporn Supasitthumrong, M.D., Ph.D.^a^, Chavit Tunvirachaisakul, M.D., Ph.D.^a^, Ali Abbas Abo Algon, MS.c ^c^, Hussein K. Al-Hakeim, Ph.D.^d^, Michael Maes, M.D., Ph.D.^a,e,f^

^a^ Department of Psychiatry, Faculty of Medicine, Chulalongkorn University, Bangkok, Thailand.

^b^ Medical Laboratory Technology Department, College of Medical Technology, The Islamic University, Najaf, Iraq.

^c^ Iraqi Education Ministry- Najaf- Iraq.

^d^ Department of Chemistry, College of Science, University of Kufa, Kufa, Iraq.

^e^ Department of Psychiatry, Medical University of Plovdiv, Plovdiv, Bulgaria.

^f^ Department of Psychiatry, IMPACT Strategic Research Centre, Deakin University, Geelong, Victoria, Australia.


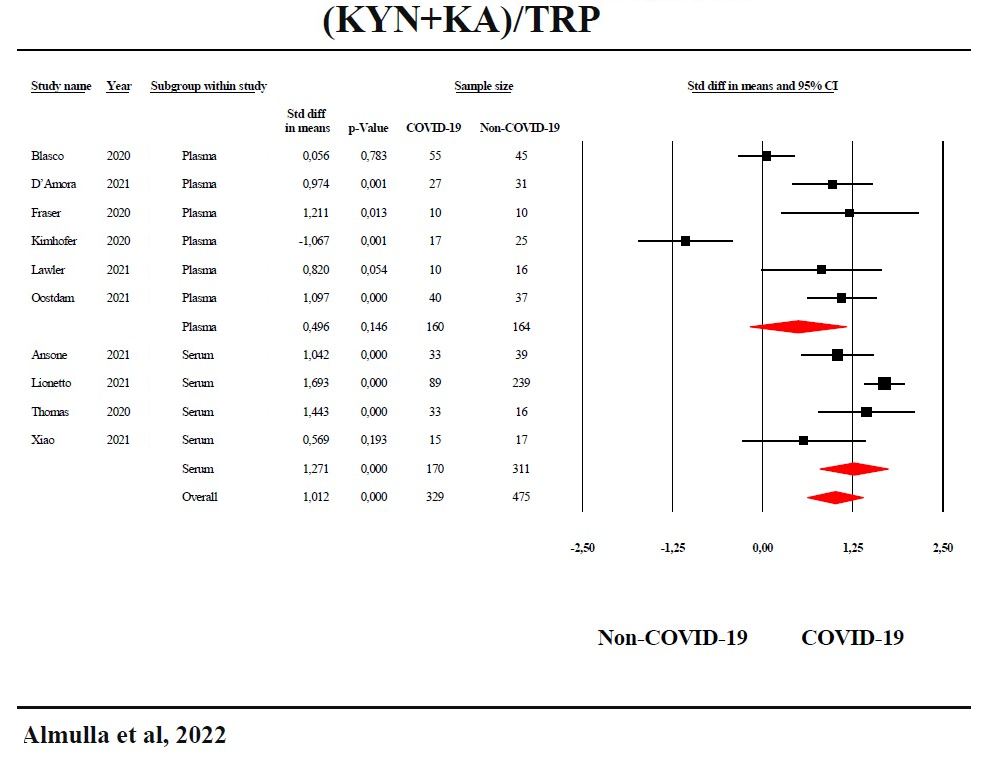


**Figure 1:** Forest plot with the results of a meta-analysis performed on the kynurenine + kynurenic acid / tryptophan (KYN+KA)/TRP ratio in COVID-19 patients versus non-COVID-19 controls.


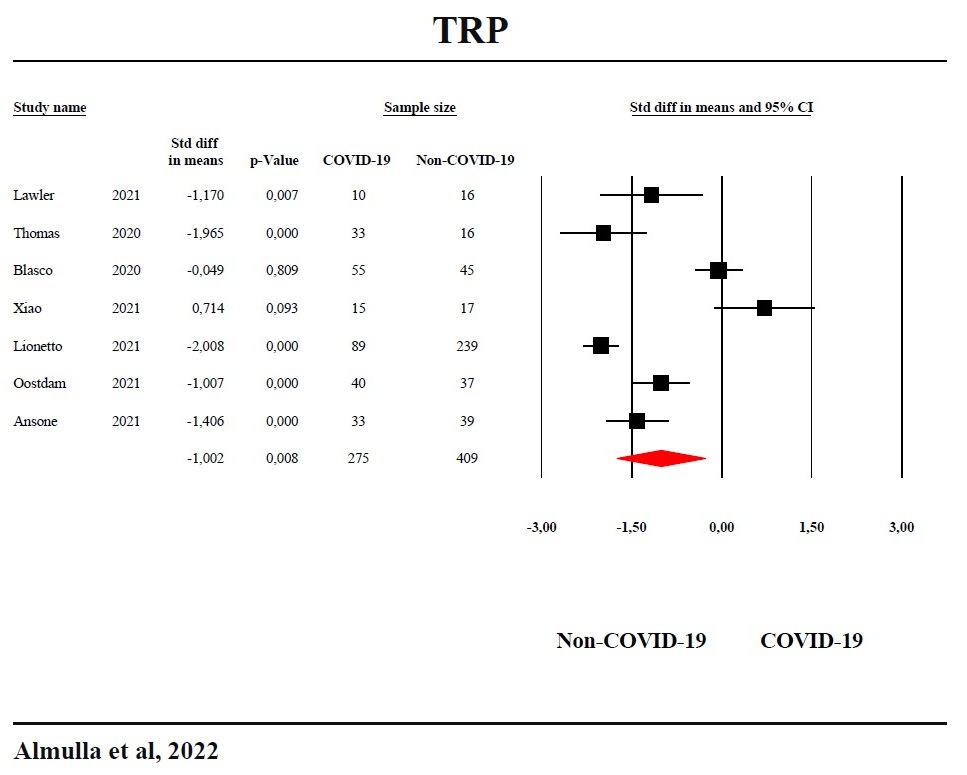


**Figure 2:** Forest plot with the results of the meta-analysis performed on tryptophan (TRP) in COVID-19 patients versus non-COVID-19 controls.


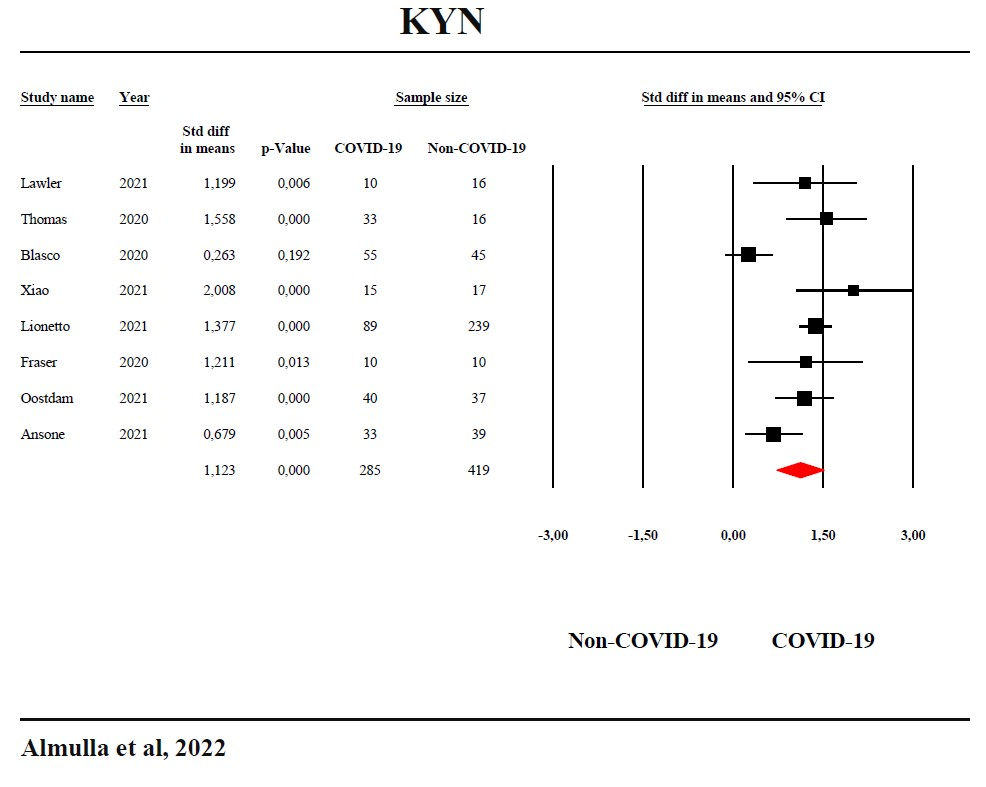


**Figure 3:** Forest plot with the results of a meta-analysis performed on kynurenine (KYN) in COVID-19 patients versus non-COVID-19 controls.


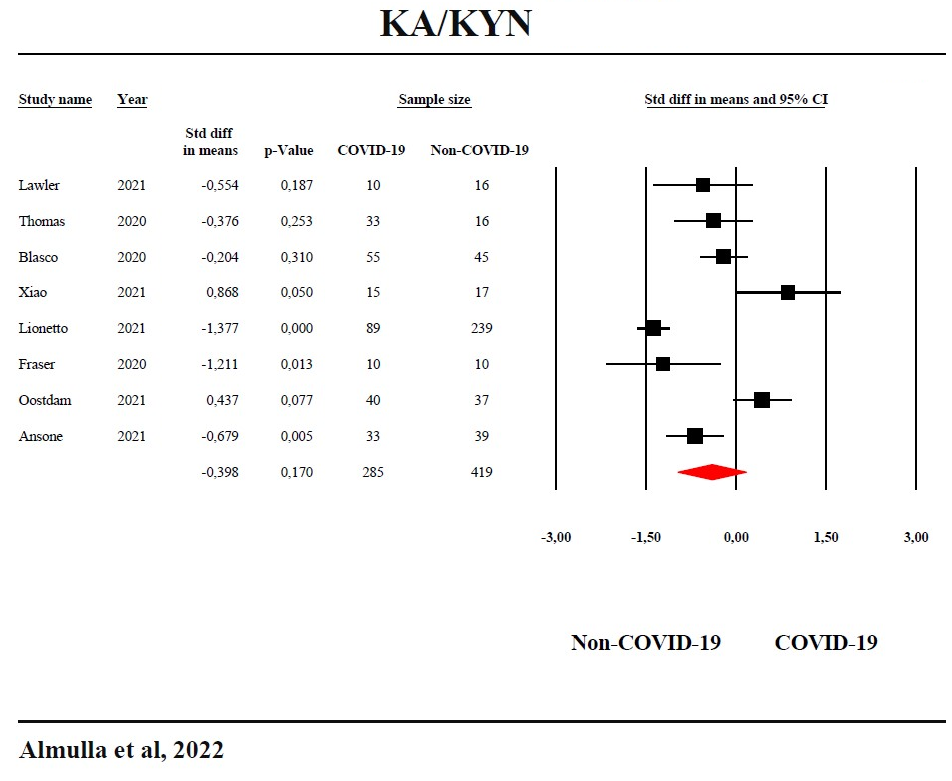


**Figure 4:** Forest plot with the results of a meta-analysis performed on the kynurenic acid / kynurenine (KA/KYN) ratio in COVID-19 patients versus non-COVID-19 controls.


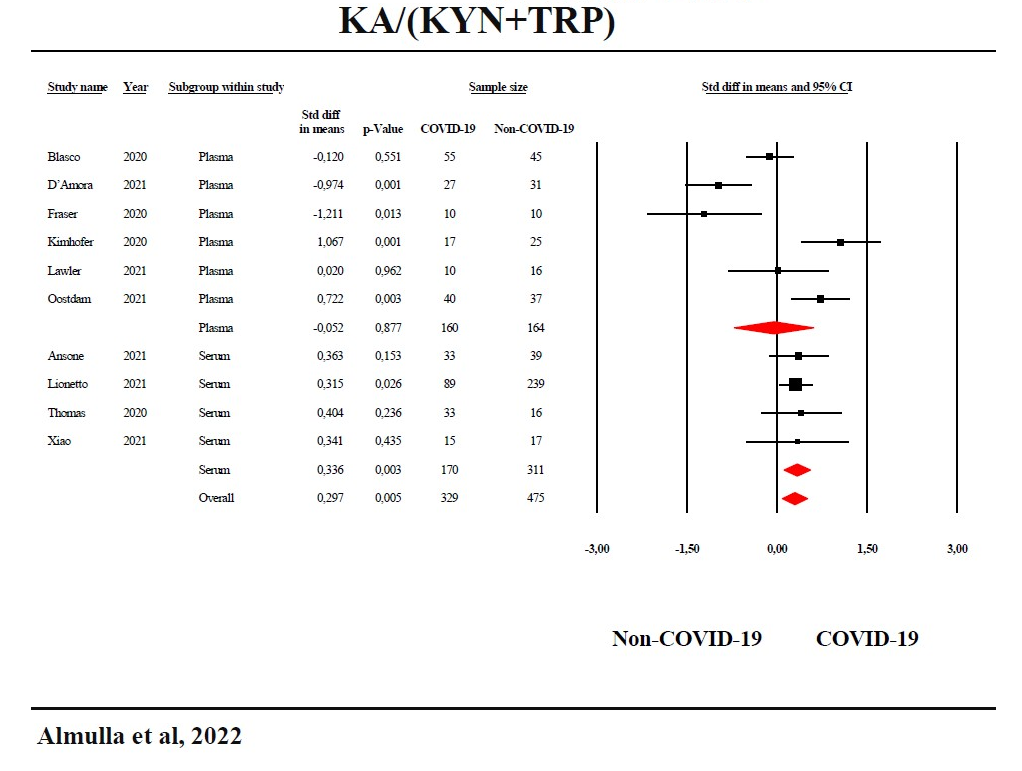


**Figure 5:** Forest plot with the results of the meta-analysis performed on the kynurenic acid / kynurenine + tryptophan (KA/KYN+TRP) ratio in COVID-19 patients versus non-COVID-19 controls.


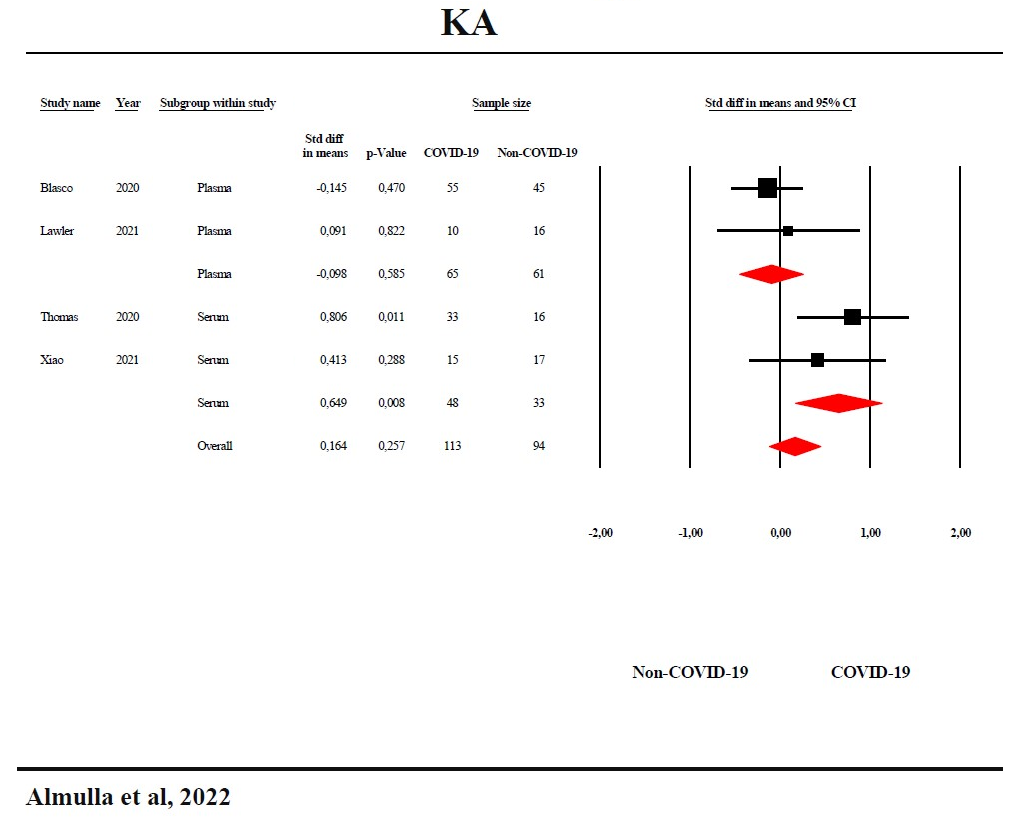


**Figure 6:** Forest plot with the results of a meta-analysis performed on kynurenic acid (KA) in COVID-19 patients versus non-COVID-19 controls.


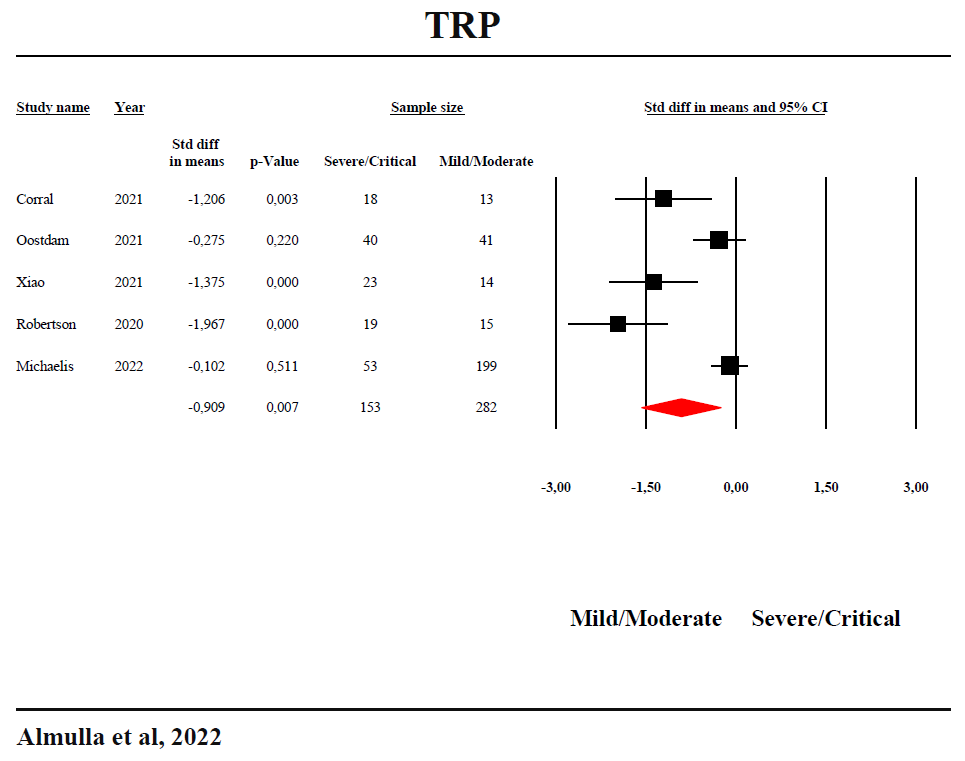


**Figure 7:** Forest plot with the results of the meta-analysis performed on tryptophan (TRP) in severe/critical COVID-19 versus mild/moderate COVID-19.


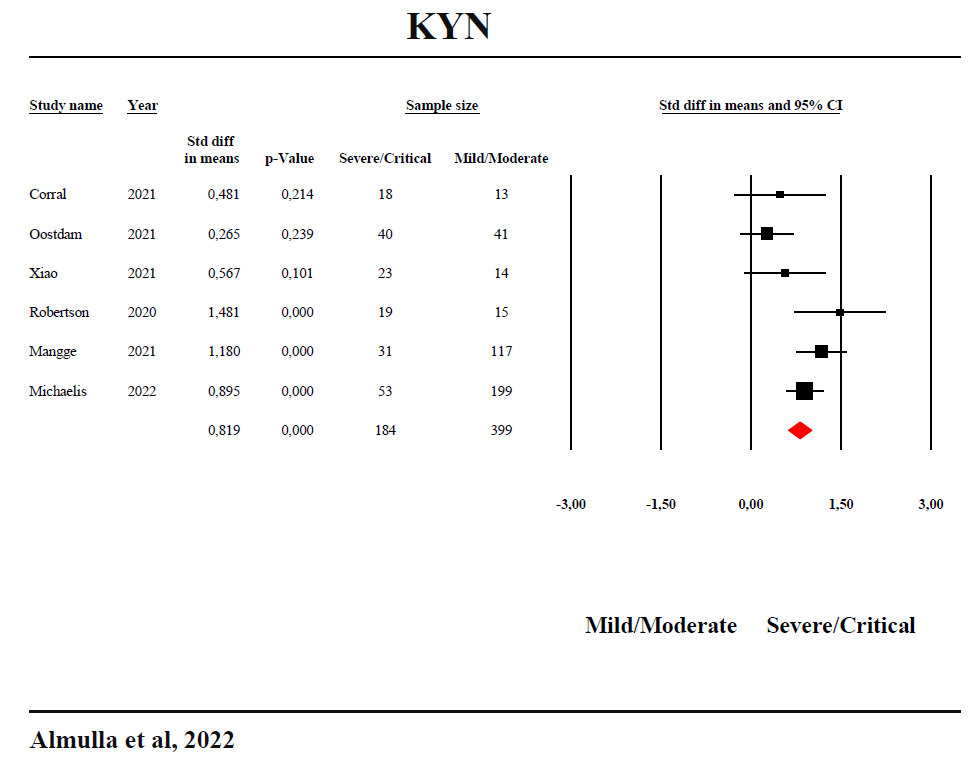


**Figure 8:** Forest plot with the results of a meta-analysis performed on kynurenine (KYN) in severe/critical COVID-19 versus mild/moderate COVID-19.
